# Functional and Morphological Responses to Fluocinolone Acetonide 0.19 mg in Noninfectious Uveitic Macular Edema Evaluated as the Area-Under-the-Curve

**DOI:** 10.1089/jop.2023.0027

**Published:** 2023-09-15

**Authors:** Lucy Joanne Kessler, Gerd Uwe Auffarth, Ramin Khoramnia

**Affiliations:** Department of Ophthalmology, University of Heidelberg, Heidelberg, Germany.

**Keywords:** drug response, ocular implant, fluocinolone acetonide, macular edema, uveitis, optical coherence tomography biomarkers

## Abstract

**Purpose:**

This study investigated the impact of baseline clinical and optical coherence tomography (OCT) factors on the response to a 0.19-mg fluocinolone acetonide (FAc) implant in patients with noninfectious uveitic macular edema evaluated by the area under the curve over 24 months.

**Methods:**

A retrospective study was conducted of eyes of patients with noninfectious uveitic macular edema undergoing FAc treatment, with follow-up from baseline to 24 months. The area under the curve (AUC) of best-corrected visual acuity (BCVA) and the central macular thickness (CMT) were calculated using the trapezoidal rule. Clinical and OCT data at the time of FAc administration were collected, and associations with AUC of BCVA and CMT changes were investigated.

**Results:**

Twenty-three patients were enrolled. BCVA and CMT significantly improved after FAc implantation (*P* < 0.05). AUC_BCVA_ and AUC_CMT_ were 0.41 ± 0.33 logarithm of minimal angle of resolution/6 months and 320.15 ± 321.64 μm/6 months, respectively. Better baseline BCVA (coefficient [coef.] = 0.83, *P* < 0.001) and macular thickness reduction after FAc administration (coef. = −0.0001, *P* < 0.05) were associated with better BCVA after FAc treatment. In contrast, baseline OCT biomarkers such as ellipsoid zone reflectivity and choroidal vascularity index, sex, or disease duration before FAc injection showed no correlation with AUC_BCVA_ and AUC_CMT_ (*P* > 0.05). The younger the patient at the time of FAc injection, the greater the reduction in CMT (coef. = 1.76, *P* < 0.05).

**Conclusions:**

Among all clinical and morphological baseline factors, Baseline BCVA was the strongest predictor for AUC_BCVA_, while no association with baseline OCT features was observed. Overall, improvement of BCVA and CMT after FAc injection was maintained over 24 months.

This study is registered in the German Clinical Trials Register under the DRKS-ID: DRKS00024399.

## Introduction

Treatment options for noninfectious uveitis (NIU) range from systemic therapy with immunosuppressive or disease-modifying antirheumatic drugs to local corticosteroid (CS) therapy or a combination of both.^1–3^ CSs are an important part of the treatment of uveitis as CSs can not only provide rapid control of acute inflammation but can also be applied as a maintenance therapy for sustained disease control over months and years.^4,5^

However, systemic CSs can cause a wide range of adverse effects and increase the risk for cardiovascular diseases such as hypertension or diabetes mellitus with long-term use.^6^ Consequently, treatment should be as precisely targeted to the affected tissue as possible to improve the efficacy and side effect profile. Applying CSs as an intravitreal sustained drug delivery system can reduce systemic drug dosage for ocular disease control and enhance patient compliance with lower injection frequency at the same time.^7^

In several European countries, a dose of 0.19 mg of fluocinolone acetonide (FAc; Iluvien; Alimera Sciences Inc., Alpharetta, GA, USA) is approved for treatment of NIU affecting the posterior segment of the eye.^8^ The implant consists of a nonbiodegradable polymer with a length of 3.5 mm with a steady low-dose release (0.2 μg/day) of FAc for up to 36 months.^7,9^ The implant is injected into the vitreous cavity with a preloaded injection pen and does not require any surgical procedure for placement.

The overall drug exposure over time can be assessed by the area under the curve (AUC).^10^ In general, it can also be applied to evaluate changes in parameters such as visual acuity changes, which reflect the pharmacodynamic responses to the drug.^11^ Notably, in contrast to single time point measurements, considering an integral over time is a more accurate estimate for the overall exposure to the drug as it accounts for the variation of the parameter over all measurements of the entire observation time.

Two recent publications, in 2020 and 2022, have reported AUC analysis of the efficacy of FAc in diabetic macular edema.^12,13^ AUC of visual acuity in NIU has been explored in a limited number of prior studies, but those reports did not include optical coherence tomography (OCT) biomarkers.^14^ Previously, we reported adverse effects such as cataract development, dysregulation of intraocular pressure, and ocular inflammation grading in patients with NIU who received FAc implants.^7^

Including OCT biomarkers such as the ellipsoid zone reflectivity ratio (EZR) and choroidal vascularity index (CVI) can contribute to the understanding of the correlation between morphological changes and visual function. There are three hyper-reflective bands in a healthy retina in the OCT: the external limiting membrane (ELM), the ellipsoid zone (EZ), and the retinal pigment epithelium (RPE), which is the most hyper-reflective band among the three structures.

The hyper-reflectivity of the EZ results from the light scattering properties of the high mitochondrial density in the inner segments of photoreceptors, thus indicating the vitality of photoreceptors.^15^ The EZR can be calculated as the reflectivity of EZ compared with the ELM or RPE, alternatively. In this study, we chose to calculate the EUZ as a reflectivity ratio to RPE because the ELM can be hard to distinguish in pathological retinas, while RPE remains mostly detectable given its high reflectivity.

It has been shown to correlate closely with the vitality and function of photoreceptors in retinal diseases such as diabetic macular edema.^16^ The CVI is a novel OCT biomarker to monitor uveitis activity with involvement of the posterior segment.^17^ The CVI represents the percentage of the vascular area of the total choroidal area in an OCT B-scan. Elevated CVI, which indicates higher blood flow within the choroid, has been reported in the active inflammatory state in NIU, and alterations of CVI during CS therapy were observed in noninfectious uveitic macular edema.^9,18^

Hence, in this study, we conducted an AUC analysis of clinical and morphological OCT parameters after FAc implantation in NIU for up to 24 months. As a secondary analysis, factors associated with the AUC of visual acuity and central macular thickness (CMT) were examined.

## Methods

This study was conducted according to the guidelines of the Declaration of Helsinki and Institutional Review Board approval was obtained (protocol code: S-644/2020, date of approval: 09.10.2020). Informed consent was obtained from all subjects involved in the study.

### Study patients

This was a retrospective study of (one eye per patient) 23 patients who had a history of at least 1 year of NIU with macular edema requiring local treatment (topical, periocular, or intravitreal), as defined by the Standardization of Uveitis Nomenclature Working Group.^19^ In cases of bilateral implantation of FAc, we chose the eye with the longer follow-up time. FAc implantation was administered at the physician's discretion.

In general, patients who received FAc implants were those who showed a good response to previous CS injection [ie, periocular triamcinolone acetonide or dexamethasone implant (Ozurdex, Allergan, Inc., Irvine, CA, USA)], but who required frequent injections. After FAc injection, verification of its position was done by slit-lamp biomicroscopy. FAc retreatment with CS after FAc implantation was administered in case of recurrent uveitic macular edema (worsening of macular edema or the onset of macular edema).

Patients with FAc implantation with a follow-up time of <6 months were excluded. Other exclusion criteria were the (absolute) spherical equivalent of more than 6 diopters, patients aged <18 years, and concomitant retinal diseases associated with macular edema.

### Data collection

The electronic charts of all study patients who received FAc between January 2014 and January 2020 were reviewed. Clinical and OCT data were reviewed at baseline and 6, 12, 18, and 24 months after FAc implantation. At each visit, patients completed measurement of best-corrected VA, tonometry, slit-lamp biomicroscopy, and indirect funduscopy performed by an experienced ophthalmologist specializing in uveitic diseases.

OCT images were obtained at each visit using Spectralis spectral-domain OCT (Heidelberg Engineering GmbH, Heidelberg, Germany). CVI and EZR were quantified as previously reported.^9^ Fiji software, version 2.1.0/1.53c (US National Institutes of Health, Bethesda, MD, USA; https://imagej.net/software/fiji), was used for all image analyses.

### Statistical analysis

Snellen VA was converted to logarithm of minimal angle of resolution (logMAR) for statistical calculation. For descriptive analysis, categorical data are presented as frequency and percentage (*n*; %); continuous data are shown as means with standard deviations. Statistical analysis and visualization were performed with the open-source programming language, Python (www.python.org, version 3.11.1).

Spearman's rho (*ρ*) was used for correlation analysis. Baseline factors associated with best-corrected visual acuity (BCVA) and CMT changes, defined as AUC_BCVA_ and AUC_CMT_, respectively, from baseline to month 24 were identified by ordinary least square regression. Intercorrelation between independent variables that potentially can lead to model overfitting was evaluated with variation inflation factor testing, and no regression models were run with variation inflation factor over 3. Variation inflation factor varied between 1.09 and 1.15.

Due to the small sample size, we deliberately chose a small number of covariates to avoid inflating the model. The Akaike information criterion was used to compare between models and choose the best fitting model. The AUC was measured using the trapezoidal rule. Missing longitudinal data were imputed with the nearest available observation. A two-sided *P* < 0.05 was considered a statistically significant difference.

## Results

### Baseline clinical characteristics

At the time of FAc injection, study patients had a mean age of 58.17 ± 15.40 years (range 25–86). Thirty percent (*n* = 7) were male, and the right eye was studied in 39% (*n* = 9) of cases. Patients were switched to FAc after a mean of 9.03 ± 5.92 years (range: 1.98–24.02) of disease duration. All eyes had received local CS treatments before FAc implantation. For 13 patients (56%) who completed the 2 years of follow-up, clinical and OCT data were complete at all observation time points.

**FIG. 1.**
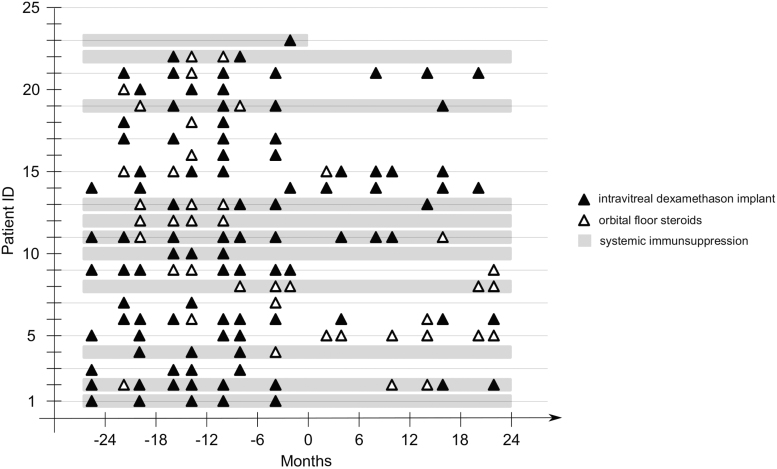
Overview of local and systemic therapy approaches before and after FAc implantation. Notably, FAc implantation enabled reduction of local CS injections in most patients. Some patients did not need adjunct CS injections after FAc implantation for up to 2 years, that is, patients 1, 3, and 4. CS, corticosteroid; FAc, fluocinolone acetonide.

All other patients were followed until month 18 after FAc implantation. Patients' characteristics and baseline OCT features are described in Table 1. Local and systemic therapy approaches 2 years before and after FAc implantation are shown in Fig. 1. Before FAc implantation (baseline/0 months), most patients received multiple intravitreal dexamethasone injections (Ozurdex) or periocular orbital floor triamcinolone acetonide injections as local therapy that was frequently applied every 3 to 6 months.

**Table 1.** Patients' Characteristics

Demographic characteristics	*n *(%) or mean (± standard deviation)
Number of patients (one eye per patient)	23
Right eye as the study eye	9 (39)
Age at FAc implantation (years)	58.17 ± 15.40
Male	7 (30)
Disease duration until FAc implantation (years)	9.03 ± 5.92
Etiology of noninfectious uveitis	
Idiopathic	12 (52)
Sarcoidosis	3 (12)
Birdshot retinopathy	2 (9)
Ocular tuberculosis (noninfectious when treated)	2 (9)
Vogt–Koyanagi–Harada syndrome	1 (4.5)
Acute zonal outer occult retinopathy	1 (4.5)
Multiple sclerosis	2 (9)
Pseudophakia	20 (87)
Vitrectomized eyes	3 (13)
Previous dexamethasone implant (Ozurdex)^a^	21 (91)
Number of dexamethasone implants^a^	3.30 ± 1.80
Previous periocular local CSs (triamcinolone acetonide)^a^	16 (70)
Number of periocular local CSs^a^	1.13 ± 0.92
Systemic medication at baseline	
Systemic CSs	3 (13)
Immune modulatory drugs other than CSs	4 (17)
Combination of both	4 (17)
No systemic medication	12 (52)
CVI at baseline	0.73 ± 0.04
EZR at baseline	0.79 ± 0.16
CMT at baseline	401.57 ± 156.93

^a^ Within 2 years (1.89 ± 0.33 years) before FAc implantation.

CMT, central macular thickness; CSs, corticosteroid; CVI, choroidal vascularity index; EZR, ellipsoid zone reflectivity ratio; FAc, fluocinolone acetonide.

Some patients were under systemic immunosuppressive therapy with prednisolone, mycophenolic acid, or cyclosporine for disease control. In most patients, the frequency and amount of local CS injections could be reduced after FAc implantation. The response to FAc varied from no adjunct need for any local steroid injections for 2 years after FAc implantation, as seen in patients 1, 3, and 4, to continued local CS therapy, as observed in patients 11, 15, and 21.

### Visual acuity and CMT changes over time

**FIG. 2.**
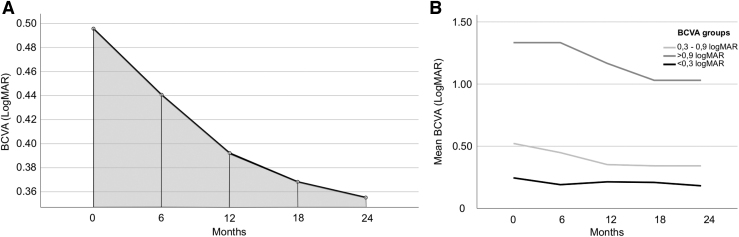
AUC_BCVA_ calculated using the trapezoidal rule and mean BCVA changes stratified according to baseline BCVA. **(A)** BCVA changes from baseline to 24 months. Measurements of each time point are plotted as a line graph and the AUC represents the summation of all trapezoidal subintervals under the plotted line (*gray area*). **(B)** Worse BCVA at baseline results in worse final visual outcome at month 24. AUC, area under the curve; BCVA, best-corrected visual acuity; logMAR, logarithm of minimal angle of resolution.

Overall, BCVA improved after FAc implantation, from 0.50 ± 0.36 logMAR at baseline to 0.36 ± 0.30 logMAR at month 24 (*P* = 0.0008) (Fig. 2A). The mean AUC_BCVA_ was 0.41 ± 0.33 logMAR/6 months (range: 0.11–1.30). Patients with worse baseline BCVA had greater visual gain up to 2 years after FAc, but their visual outcome over time remained worse than patients with better baseline BCVA (Fig. 2B).

**FIG. 3.**
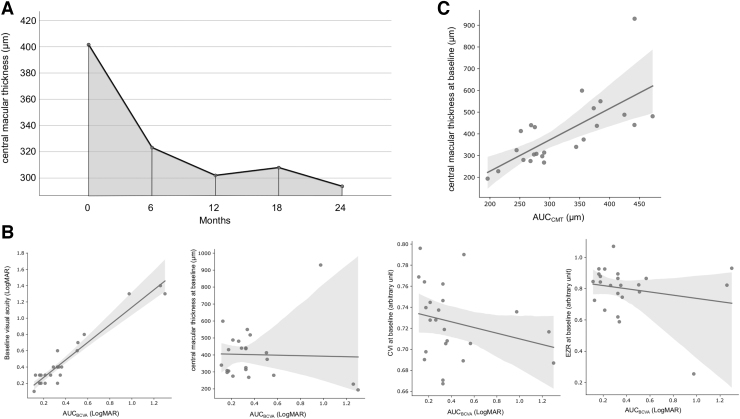
AUC_CMT_ calculated using the trapezoidal rule and correlation between baseline parameters and AUC_BCVA_. **(A)** CMT changes from baseline to 24 months. Measurements of each time point are plotted as a line graph and the AUC represents the summation of all trapezoidal subintervals under the plotted line (*gray area*). **(B)** Linear correlation between baseline parameters (BCVA, CRT, CVI, and EZR) and AUC_BCVA_/6 months. The regression coefficient was calculated with Spearman's rank correlation. **(C)** CMT at baseline correlated with AUC_CMT_. CMT, central macular thickness; CRT, central retinal thickness; CVI, choroidal vascularity index; EZR, ellipsoid zone reflectivity ratio.

The mean CMT decreased from 401.60 ± 32.72 μm at baseline to 293.65 ± 14.33 μm after 24 months (*P* = 0.03) (Fig. 3A). The mean AUC_CMT_ was 320.15 ± 321.64 μm/6 months (range: 296–471 μm). AUC_BCVA_ correlated with baseline BCVA (*ρ* = 0.85; *P* < 0.001), but not EZR (*ρ* = 0.18; *P* = 0.40), CVI (*ρ* = 0.37; *P* = 0.08), and CMT (*ρ* = 0.14; *P* = 0.52) (Fig. 3B) at baseline.

CMT at baseline correlated with AUC_CMT_ (*ρ* = 0.75; *P* < 0.001) (Fig. 3C). AUC_BCVA_ and AUC_CMT_ showed no correlation (*P* > 0.05). Changes in EZR and CVI before and after FAc implantation are shown in the Supplementary Figure S1. After FAc injection, there is a tendency of improved CVI (lower CVI) and EZR (higher EZR). However, the difference between baseline and 2 years after FAc implantation was not significant for both OCT biomarkers (EZR: *P* = 0.078, CVI: *P* = 0.209).

### Factors associated with AUC_BCVA_ and AUC_CMT_

Better baseline BCVA (coefficient [coef.] = 0.83; 95% confidence interval [CI] = 0.73–0.93, *P* < 0.001) and macular thickness reduction after FAc administration (expressed as AUC_CMT_, coef. = −0.0001; 95% CI = 0.00–2.5 × 10^−8^, *P* < 0.05) were associated with better BCVA after FAc treatment. In contrast, age at FAc administration, disease duration, previous treatment received, history of vitrectomy, and uveitis location were not significantly associated with AUC_BCVA_ (*P* > 0.05).

CVI and EZR at baseline and their AUCs showed no correlation (*P* > 0.05). Regression models, including baseline BCVA, showed an adjusted *R*^2^ of 0.92, while models without baseline BCVA had a much lower adjusted *R*^2^ of ∼0.13. Thus, baseline BCVA was the strongest predictor for AUC_BCVA_.

The AUC_CMT_ was positively correlated with the CMT at baseline (coef. = 0.40; 95% CI 0.23–0.57; *P* < 0.001) and the patient's age at FAc injection (coef. = 1.76; 95% CI = 0.05–3.47; *P* < 0.05), while CVI and EZR at baseline, disease duration, and AUC_BCVA_ were not significantly associated with AUC_CMT_.

## Discussion

The therapeutic effect of the 700-μg biodegradable dexamethasone implant, Ozurdex, has been verified in numerous studies for diabetic macular edema and uveitis with involvement of the posterior segment.^20,21^ All patients in this retrospective study also received dexamethasone implants before receiving the FAc implant, Iluvien. The advantage of the FAc implant is found in the different pharmacokinetics of both implants.

While the dexamethasone implant represents a shorter acting agent with a high drug release rate in the first 2 months and low-dose release for a further 4 months, FAc was designed for long-term release of low-dose CSs in a nearly zero-order kinetic over a 36-month period.^22^ Therefore, the FAc implant is suitable for long-term management of chronic inflammatory diseases in the eye.

Previous studies reported that continued release of low-dose CS led to better anatomical outcomes such as less fluctuation in central retinal thickness.^23,24^ As shown in our study, FAc implantation reduced the frequency and amount of needed CS in the follow-up, thus reducing the treatment burden for patients. Risks of intravitreal injections, such as endophthalmitis, scleral scarring, or postoperative hypotension, can consequently be avoided or minimalized.

In comparison with Retisert (Bausch & Lomb/Valeant, Bridgewater, NJ, USA), a 590-μg FAc implant, which needs to be fixed to the sclera by a surgical procedure and has inherent risks such as scleral melt, retinal detachment, and choroidal bleeding, administration of the FAc implant, Iluvien, by an injection pen is safer, requires no special surgical skills, and bears less risk for the patient.

The functional and anatomic effectiveness of the FAc implant in NIU has been previously described.^7,25,26^ In accordance, this study demonstrated improvement in BCVA and CMT under the continuous low-dose release of CSs. Our results underlined baseline BCVA as a predictor for AUC_BCVA_ over a mean of 2 years, while patients' demographic features such as age and disease duration were not significantly correlated with the functional outcome.

Patients with worse baseline VA experienced greater BCVA improvement, but their final visual outcome was inferior to those with better baseline BCVA. A similar relationship between baseline BCVA and functional outcome has been observed in patients with macular edema who received antivascular endothelial growth factor (VEGF) therapy or CS injections for retinal diseases such as age-related macular degeneration or diabetic macular edema.^27,28^ These results emphasize baseline BCVA as a major prognostic factor for the overall outcome and importance of a timely start of treatment.

Likewise, eyes with lower CMT at baseline and younger patients are more likely to have a better morphological response (expressed as AUC_CMT_) in this study. Older age has been linked as a negative predictor for therapy response in treatment with anti-VEGF or dexamethasone agents.^29,30^ However, so far, this association has not been observed in noninfectious uveitic macular edema treated with sustained CS implants.

The average drug effect over time was calculated with the AUC method, which has been used in randomized controlled studies of diabetic macular edema.^13,31^ However, published analysis of AUCs in noninfectious uveitic macular edema is scarce. Battista et al.^14^ performed a retrospective study with 10 eyes from seven patients for 1 year after FAc administration and found that visual acuity and severity of macular thickness at baseline were the main determinants of drug response; but OCT biomarkers were excluded from their analysis.

In contrast, Cicinelli et al. examined the AUC analysis in diabetic macular edema treated with FAc and concluded that ellipsoid zone integrity at baseline was correlated with better visual outcome.^12^ In our study, neither CVI nor EZR at baseline was a predictor for long-term functional and morphological responses. This could be not only due to the distinct pathology of different ocular diseases but also because of the heterogeneity of underlying uveitic diseases in our cohort.

The role of OCT biomarkers such as CVI and EZR as predictors for treatment response has been investigated in various retinal diseases with macular edema. CVI describes the ratio between vascular and stromal areas in the choroid, which reflects abnormal choroidal flow not only in several uveitic entities but also in diabetic retinopathy.^32,33^ We previously demonstrated that a greater CVI reduction within 6 months after FAc implantation was inversely associated with the need for additional local CSs,^9^ but CVI does not seem to be associated with functional or morphological outcome in this cohort.

The strong association between EZR and visual acuity has been noted by several authors for retinal and uveitic diseases and confirmed in a small number of uveitis studies.^34,35^ Nevertheless, we found no direct association with visual outcome in this study. Possibly, the small sample size and wide range of included baseline visual acuity led to a selection bias.

Moreover, there are reports of a lack of association between gender, history of vitrectomy, and functional outcome in diabetic macular edema treated with FAc.^21,36,37^ Our study confirmed these observations in noninfectious uveitic macular edema.

The limitation of this study lies in the retrospective design, small sample size, and heterogenicity of the underlying uveitic diseases such as idiopathic, multiple sclerosis-related, and granulomatous uveitis. Furthermore, it would have been desirable to evaluate the outcome beyond 2 years after FAc implantation as the implant has an effective duration of up to 3 years.^4^

As data between 2 and 3 years after FAc implantation were not available for most patients, we chose not to impute the data to avoid the shortcomings associated with imputation. In addition, due to the small sample size, the regression analysis might be underpowered. Nevertheless, this study sheds light on factors associated with functional and morphological drug responses in patients with NIU and it can provide a basis for future research.

In conclusion, our study confirmed results from previous reports and added a complement analysis of OCT biomarkers, namely EZR and CVI, and their relationship with the functional outcome. To our best knowledge, no such analysis has been conducted in patients with NIU. Our results are based on long-term experiences with the FAc implant, Iluvien, since its approval by the European Medicines Agency in 2015.

These results may serve as a reference for the recently approved (in 2018) intravitreal FAc implant, Yutiq (EyePoint Pharmaceuticals, Inc., Watertown, MA, USA), which is available in the United States. Yutiq has almost the same total amount of FAc as Iluvien and pharmacokinetics can be expected to be similar. In future, studies with a larger cohort and longer follow-up are needed to confirm these results.

## Supplementary Material

Supplemental data
