# Supplementary material for: Functional and Morphological Responses to Fluocinolone Acetonide 0.19 mg in Noninfectious Uveitic Macular Edema Evaluated as the Area-Under-the-Curve
Source: J Ocul Pharmacol Ther. 2023 Sep 15;39(7):449–55. doi: 10.1089/jop.2023.0027 (PMC10516239; doi:10.1089/jop.2023.0027)
Supplement: Supplementary file 1 [file Suppl_FigureS1.docx]

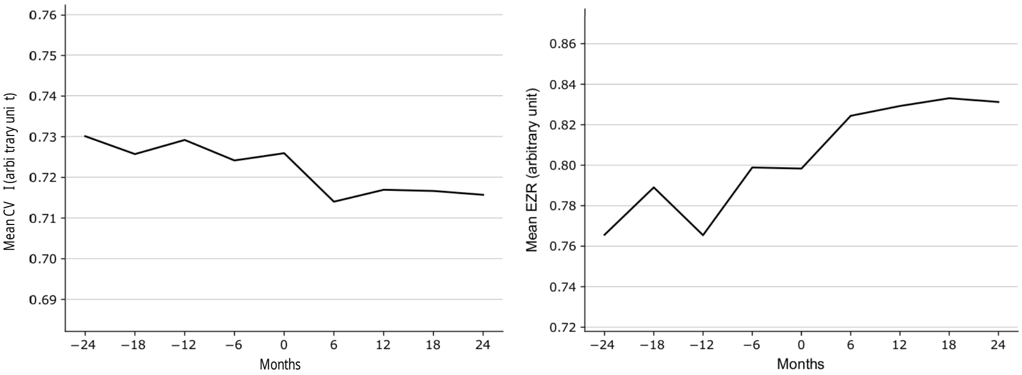

Mean CVI (arbitrary unit)
0.76
0.75
0.74
0.73
0.72
0.71
0.70
0.69
−24 −18 −12 −6 0 6 12 18 24
Months
Mean EZR (arbitrary unit)
0.86
0.84
0.82
0.80
0.78
0.76
0.74
0.72
−24 −18 −12 −6 0 6 12 18 24
Months
